# Work and Worry

**Published:** 1879-09

**Authors:** 


### WORK AND WORRY.

"STUDYING too hard," "over-worked," are charged with many deaths, but not wisely. Trouble kills; it is a very rare thing for a man to think himself to death, unless connected with something more or less distressing. Study is a bliss to the student; he had rather study than eat; the sound of the dinner bell is always unwelcome; the greatest students in moral philosophy, and divinity, and physics (not physic) have lived long and worked efficiently to fourscore and beyond. Thought is to the brain what exercise is to the physical constitution; it keeps the channels of life clear, the blood vessels unobstructed, and the vital fluid courses along them, distributing newness of life and vigor of action to the latest hour of existence, while the want of thought brings stagnation to the circulation, and causes man to drivel and sleep in old age—dead as to everything except to eating, and dozing, and hovering over the fire.

Men may study ever so hard, and after fifty may study with comfort and advantage for five, ten and fifteen hours, day after day; and if the studies are pleasurable they promote the general well-being of the system, both physical and mental, only if abundant sleep is had, with a regular supply of simple and nourishing food, sitting down to meals in pleasant moods, and allowing a good half hour before study is resumed. Many of our literary men die prematurely, not from over study, but from depressing mental states and irregular or excessive eating and drinking.

It is haste, rather than steady, continuous labor of body or mind which hurries multitudes to their graves scores of years before their time. With all haste there is impatience, solicitude, worry. The fastest trains, the fleetest steamers, the first trotters everywhere command premiums. To save time "night boats" are patronized, breakfast is bolted, the morning paper read on the cars, and everything is done under high pressure. But just as certainly as a bank balance, rapidly drawn upon, melts away before it was expected, so does this reserve of vital stamina disappear—is used up, and the man dies in his prime, at the very moment, often, when he had just got into a position where he could afford to enjoy himself.

